# Effectiveness of external factors to reduce the risk of dehydration in older people living in residential care: a systematic review

**DOI:** 10.1186/1472-6963-14-S2-P11

**Published:** 2014-07-07

**Authors:** Diane Bunn, Florence Jimoh, Stephanie Howard Wilsher, Lee Hooper

**Affiliations:** 1 Norwich Medical School, University of East Anglia, Norwich, UK; 2 Faculty of Social Sciences, University of East Anglia, Norwich, UK

## Background

Water-loss dehydration, when fluid output exceeds fluid intake, is prevalent in older people living in care homes, due to declining kidney function and poorer thirst sensation exacerbated by increasing comorbidities and impaired mental and physical capacities to obtain a drink. As dehydration is associated with poor health outcomes, effective prevention strategies will improve quality of life.

## Methods

A systematic review, following the Cochrane Collaboration’s guidelines (http://www.cochrane.org/) aimed to identify effective interventions and modifiable factors which improved hydration status and/or fluid intake in older people (≥ 65 years) living in residential care who could drink orally (http://www.crd.york.ac.uk/Prospero/display_ record,asp?ID=CRD42OI 2003100).

Thirteen electronic databases were searched from inception until 30^th^ September 2013 in all languages, with additional searches of key authors, reference lists of reviews and included papers. Using predetermined criteria, two reviewers independently selected studies for inclusion, abstracted data and assessed validity.

## Results

Searches identified 4328 titles and abstracts after 856 duplicates were removed. 325 full-text papers were obtained and 23 studies included (nineteen intervention and 4 observational studies) from 7 countries.

A wide range of interventions and exposures were identified, but the lack of suitable ways of accurately assessing fluid intake and/ or dehydration, as well as paucity of randomisation and allocation concealment resulted in the efficacy of many strategies being unproven.

Two observational studies with low risk of bias conducted secondary analyses demonstrating that for-profit ownership in Canada was associated with higher rates of hospital admission for dehydration compared to not-for-profit homes, whilst in the United States (US) there was no difference. Studies at higher risk of bias reported lower rates of dehydration following implementation of the Resident Assessment Instrument (US), eating in a less-institutionalised setting (US, UK), eating with others (US) or use of high-contrast red tableware compared with white (US). No effect was seen for number of residents present in the dining-room, allocation of a permanent seating position, noise level, position of feeding assistants, type of thickening agents used, staff training or staff: resident ratios (Canada, Ireland, US). However, these studies were small so useful effects may have been missed.

Multi-component interventions in Germany, Japan, Taiwan and US (including increased availability, attractiveness and choice of drinks, education, assistance with drinking and toileting) were associated with increased fluid intake when between-meals drinks rounds were introduced.

## Conclusions

There are some promising interventions to improve fluid intake in care homes, but high quality well-powered randomised controlled trials are needed to confirm their efficacy.

